# Cannabigerol Action at Cannabinoid CB_1_ and CB_2_ Receptors and at CB_1_–CB_2_ Heteroreceptor Complexes

**DOI:** 10.3389/fphar.2018.00632

**Published:** 2018-06-21

**Authors:** Gemma Navarro, Katia Varani, Irene Reyes-Resina, Verónica Sánchez de Medina, Rafael Rivas-Santisteban, Carolina Sánchez-Carnerero Callado, Fabrizio Vincenzi, Salvatore Casano, Carlos Ferreiro-Vera, Enric I. Canela, Pier Andrea Borea, Xavier Nadal, Rafael Franco

**Affiliations:** ^1^Department of Biochemistry and Physiology, Faculty of Pharmacy, University of Barcelona, Barcelona, Spain; ^2^Centro de Investigación Biomédica en Red, Enfermedades Neurodegenerativas (CIBERNED), Instituto de Salud Carlos III, Madrid, Spain; ^3^Department of Medical Sciences, Institute of Pharmacology, University of Ferrara, Ferrara, Italy; ^4^Molecular Neurobiology Laboratory, Department of Biochemistry and Molecular Biomedicine, University of Barcelona, Barcelona, Spain; ^5^Department of R&D – Extraction, Phytoplant Research S.L., Córdoba, Spain; ^6^Department of Analytical Chemistry, Phytoplant Research S.L., Córdoba, Spain; ^7^Department of Breeding and Cultivation, Phytoplant Research S.L., Córdoba, Spain

**Keywords:** cannabinoid receptor, cannabigerol, G-protein-coupled receptor, phytocannabinoid, TR-FRET, partial agonist

## Abstract

Cannabigerol (CBG) is one of the major phytocannabinoids present in *Cannabis sativa* L. that is attracting pharmacological interest because it is non-psychotropic and is abundant in some industrial hemp varieties. The aim of this work was to investigate in parallel the binding properties of CBG to cannabinoid CB_1_ (CB_1_R) and CB_2_ (CB_2_R) receptors and the effects of the compound on agonist activation of those receptors and of CB_1_–CB_2_ heteroreceptor complexes. Using [^3^H]-CP-55940, CBG competed with low micromolar *K*_i_ values the binding to CB_1_R and CB_2_R. Homogeneous binding in living cells, which is only technically possible for the CB_2_R, provided a 152 nM *K*_i_ value. Also interesting, CBG competed the binding of [^3^H]-WIN-55,212-2 to CB_2_R but not to CB_1_R (*K*_i_: 2.7 versus >30 μM). The phytocannabinoid modulated signaling mediated by receptors and receptor heteromers even at low concentrations of 0.1–1 μM. cAMP, pERK, β-arrestin recruitment and label-free assays in HEK-293T cells expressing the receptors and treated with endocannabinoids or selective agonists proved that CBG is a partial agonist of CB_2_R. The action on cells expressing heteromers was similar to that obtained in cells expressing the CB_2_R. The effect of CBG on CB_1_R was measurable but the underlying molecular mechanisms remain uncertain. The results indicate that CBG is indeed effective as regulator of endocannabinoid signaling.

## Introduction

Cannabinoid compounds bind and activate cannabinoid CB_1_ (CB_1_R) and CB_2_ (CB_2_R) receptors, which belong to the superfamily of G-protein-coupled receptors. There are many ways to classify them, but the most used distinguishes between endogenous molecules (endocannabinoids), phytocannabinoids and synthetic cannabinoids. Endocannabinoids and one of the most studied phytocannabinoids, Δ^9^-tetrahydrocannabinol (Δ^9^-THC), are agonists with more or less CB_1_R/CB_2_R selectivity. Furthermore, synthetic cannabinoids mainly act (as agonists or antagonists) by binding to the orthosteric site of receptors (Mechoulam, 2016). Indeed, there is a limited number of molecules, either synthetic or phytocannabinoids, that behave as allosteric modulators of cannabinoid receptor function.

Anandamide and 2-arachidonoyl glycerol (2-AG) are the two main endocannabinoids, being synthesized from membrane lipids and having an alkyl-amide chemical structure. They are retrograde effectors being produced in the post-synaptic neuron to act in the pre-synaptic neuron where they regulate the release of neurotransmitters (Diana and Marty, 2004).

Phytocannabinoids are phenolic terpenes biosynthesized in nature nearly exclusively in the *Cannabis sativa* L. plant. In the *Cannabis* plant, all cannabinoids are biosynthesized in the acid form, mainly Δ^9^-THCA, CBDA, etc. CBGA is the first molecule formed in the biosynthetic pathway and the substrate of Δ^9^-tetrahydrocannabinol-synthase and CBD-synthase (Fellermeier and Zenk, 1998). The pharmacologic effects of *Cannabis* components, traditionally consumed through inhalation, are attributed to the decarboxylated neutral products of above mentioned acids: Δ^9^-THC, CBD, and CBG.

Synthetic cannabinoids are very different in chemical structure. For instance, they may be indoles like WIN-55,212-2, AM-1241 or JWH-018, or phenolic, phenols lacking the pyrene ring, like CP-55,940 or HU-308. All these compounds have been used in cannabinoid research and have helped to unveil pharmacological aspects of the endocannabinoid system. It should be noted that some of these compounds have recently arrived at the streets sold as *legal highs*, thus raising Public Health concerns (Adams et al., 2017; Weinstein et al., 2017).

The endocannabinoid system is constituted by the endogenous cannabinoids, the enzymes that produce and degrade them, and by the receptors that mediate their actions. Whereas endocannabinoids consist of molecules with aliphatic structure, AEA and 2-AG, the structure of natural cannabinoids, derived from *C. sativa* L., is fairly different [see (Lu and Mackie, 2016) and references therein]. Although it is well established that one of the main active components of the plant and one of the few that are psychoactive, namely Δ^9^-THC, acts via cannabinoid receptors, there is controversy on whether these receptors mediate the action of phytocannabinoids such as CBN, CBD or CBG. As happened the last years for CBD, a new research and revision of the cannabinoid receptor pharmacology must be done with the rest of phytocannabinoids as CBG. A further phenomenon that may be considered to understand the action of molecules from *C. sativa* L. and its extracts is the fact that cannabinoid receptors may form heteromers, namely CB_1_–CB_2_ heteroreceptors, which display particular functional properties (Callén et al., 2012). It should be noted that in CNS those heteromers are mainly expressed in pallidal neurons (Lanciego et al., 2011; Sierra et al., 2015) and in activated microglia (Navarro et al., 2018a).

Cannabigerol was isolated, characterized and synthetized by the same researchers than reported the structure of the main psychotropic agent of *Cannabis*, Δ^9^-THC (Gaoni and Mechoulan, 1964). Few years later *in vivo* assays showed that CBG was non-psychoactive (Grunfeld and Edery, 1969; Mechoulam et al., 1970). The lower concentration and the lack of psychoactivity was probably the cause that CBG was shadowed by Δ^9^-THC. In fact, CBG has attracted less attention than Δ^9^-THC and even than CBD, but nowadays is gaining interest among the scientific community. Some commercial hemp varieties have CBG and CBGA as main cannabinoids and, therefore, CBG is another of the phytocannabinoids to be considered by the unregulated market of hemp oils and derivatives. As recently pointed out, the increased therapeutic potential of *C. sativa* L. components requires a more in deep understanding of the pharmacology of phytocannabinoids other than Δ^9^-THC, namely CBD, CBG, CBN, Δ^9^-THCV, Δ^8^-THC, CBC and CBDV (Turner et al., 2017).

Preliminary results using membranes from mice brain or from CHO cells expressing the human CB_2_R led to postulate that CBG could be a partial agonist at both CB_1_R and CB_2_R with *K*_i_ values in the 300–500 nM range (Gauson et al., 2007; Pertwee, 2008). The first published data on the binding of CBG to human CB_1_R and CB_2_R were provided by (Rosenthaler et al., 2014) working with [^3^H]CP-55,940 as radioligand and with preparations from Sf9 cells co-expressing one receptor and the Gαi3β1γ2 protein. The *K*_i_ values obtained in competition assays are 897 and 153 nM for CB_1_R and CB_2_R, respectively. CBG may modulate the activity of transient receptor potential channels of ankyrin type-1; however, the EC_50_ values lie in the micromolar range (De Petrocellis et al., 2008). It has been reported that CBG binds to CB_1_R (*K*_i_ = 381 nM) from mouse brain membranes and CB_2_R (*K*_i_ = 2.6 μM) from CHO cells expressing the human receptor; CBG at high concentrations (10 μM) antagonized [^35^S]GTPγS binding in mouse brain membranes treated with AEA or CP-55940 (Cascio et al., 2010). Authors also reported CBG as α_2_-adrenoceptor agonist at nanomolar levels (EC_50_ = 0.2 nM), and being also able to antagonize [^35^S]GTPγS binding upon stimulation of the 5HT_1A_ receptor by 1 μM 8-OH-DPAT (Cascio et al., 2010). Other findings indicate that CBG can act as (i) agonist/desensitizer of TRPA1 (EC_50_ = 700 nM), (ii) agonist of TRPV1 (EC_50_ = 1.3 μM) (iii) agonist of TRPV2 (EC_50_ = 1.7 μM), (iv) antagonist of TRPM8 channels (IC_50_ = 160 nM) and v) inhibitor of AEA cell uptake (*K*_i_ = 11.3 μM) (De Petrocellis et al., 2011). More recently, the PPARγ has been reported as target of the phytocannabinoid CBG (*K*_i_ = 11.7 μM) that at high concentrations, in the 10–25 μM range, may enhance the PPARγ transcriptional activity (Granja et al., 2012; Nadal et al., 2017). A recent review substantiates the complexity of the field and highlights that other players, GPR55 for instance, are also targeted by cannabinoids (Solymosi and Kofalvi, 2017).

The aim of this work was to characterize CBG pharmacology on the cannabinoid receptors using binding and measurement of different signal transduction mechanisms in living HEK-293T cells expressing human CB_1_R, CB_2_R, or CB_1_–CB_2_ heteroreceptor complexes. The results indicate that, in our experimental conditions, CBG mainly acts on CB_2_R and behaves as a partial agonist.

## Materials and Methods

### Reagents

ACEA, JWH133, and AEA were purchased from Tocris Bioscience (Bristol, United Kingdom), CBD and CBG analytical standard solutions were purchased from THCpharm (Frankfurt, DE). Concentrated (10 mM) stock solutions prepared in ethanol (CBG, ACEA, and AEA) or DMSO (JWH133 and CM-157) were stored at -20°C. In each experimental session, aliquots of concentrated solutions of compounds were thawed and conveniently diluted in the appropriate experimental solution. For non-radioactive binding assays, TLB was obtained from Cisbio Bioassays (LABMED; Codolet, France). The Tb derivative of O6-benzylguanine was synthesized by Cisbio Bioassays and is commercialized as SNAP-Lumi4-Tb (SSNPTBC; Cisbio Assays). The plasmid encoding for the SNAP-tagged human CB_2_R used for transient transfection was obtained from Cisbio Bioassays (PSNAP-CB2). CB_2_R agonist 3-[[4-[2-tert-butyl-1-(tetrahydropyran-4-ylmethyl)benzimidazol-5-yl]sulfonyl-2-pyridyl]oxy]propan-1-amine (CM-157) conjugated to a fluorescent probe was developed in collaboration with Cisbio Bioassays (Martínez-Pinilla et al., 2016).

### Cannabinoid Isolation, Purification and Analysis

Cannabidiol was purified from dried leaves and inflorescences of the *Cannabis* variety SARA (CPVO file number: 20150098), CBG from the variety AIDA (CPVO file number: 20160167) following a previously described method (Nadal, 2016) that provides compounds with >95% purity. An Agilent liquid chromatography set-up (Model 1260, Pittsburgh, PA, United States) consisting of a binary pump, a vacuum degasser, a column oven, an autosampler and a diode array detector (DAD) equipped with a 150 mm length × 2.1 mm internal diameter, 2.7 μm pore size Poroshell 120 EC-C18 column was used for the quality control of the purified cannabinoids. The analysis was performed using water and acetonitrile both containing ammonium formate 50 mM as mobile phases. Flow-rate was 0.2 mL/min and the injection volume was 3 μL. Chromatographic peaks were recorded at 210 nm. All determinations were carried out at 35°C. All samples were analyzed in duplicate. The results of each cannabinoid purity, 96.04% for CBD and 99.9% for CBG, were calculated as weight (%) versus a commercial standard from THCpharm (CBD batch n° L01258-M-1.0; CBG batch n° L01260-M-1.0).

### Radioligand Binding Assays

#### Cell Culture and Membrane Preparation

For radioligand binding experiments CHO cells, stably transfected with cDNA for human CB_1_ or CB_2_ cannabinoid receptors, were grown adherently and maintained in Ham’s F12 containing 10% fetal bovine serum, penicillin (100 U/mL), streptomycin (100 μg/mL) and geneticin (G418, 0.4 mg/mL) at 37°C in a humid atmosphere of 5% CO_2_. Membranes were prepared from cells washed with PBS and scraped off plates in ice-cold hypotonic buffer (5 mM Tris HCl, 2 mM EDTA, pH 7.4). The cell suspension was homogenized with a Polytron and then centrifuged for 30 min at 40,000 × *g*.

#### Saturation Binding Experiments

[^3^H]-CP-55940 saturation binding experiments (specific activity 169 Ci/mmol, Perkin Elmer) were performed incubating different concentrations of the radioligand (0.03 – 10 nM) in binding buffer (50 mM Tris-HCl, pH 7.4, 2.5 mM EDTA, 5 mM MgCl_2_ for CB_1_R or 50 mM Tris-HCl, pH 7.4, 1 mM EDTA, 5 mM MgCl_2_ for CB_2_R) using CHO membranes expressing the human versions of CB_1_R or CB_2_R (10 μg protein/sample) at 30°C. Non-specific binding was determined in the presence of 1 μM WIN-55,212-2. At the end of the incubation period (90 min for CB_1_R or 60 min for CB_2_R) bound and free radioactivity were separated in a cell harvester (Brandel Instruments) by filtering the assay mixture through Whatman GF/B glass fiber filters. The filter-bound radioactivity was counted in a 2810 TR liquid scintillation counter (Perkin Elmer).

[^3^H]-WIN-55,212-2 saturation binding experiments (specific activity 48 Ci/mmol, Perkin Elmer) were performed incubating different concentrations of the radioligand (0.5–100 nM for CB_1_R or 0.2–40 nM for CB_2_R) in binding buffer (50 mM Tris-HCl, pH 7.4, 1 mM EDTA, 5 mM MgCl_2_) with CB_1_R- or CB_2_R-containing CHO cell membranes (10 μg protein/sample) at 30°C. Non-specific binding was determined in the presence of 1 μM WIN-55,212-2. At the end of the incubation period (60 min) bound and free radioactivity were separated in a cell harvester (Brandel Instruments) by filtering the assay mixture through Whatman GF/B glass fiber filters. The filter-bound radioactivity was counted in a 2810 TR liquid scintillation counter (Perkin Elmer).

#### Competition Binding Experiments

[^3^H]-CP-55940 competition binding experiments were performed incubating 0.3 nM of radioligand and different concentrations of the tested compounds with membranes obtained from CHO cells expressing human CB_1_ or CB_2_ receptors (10 μg protein/sample) for 90 min (CB_1_R) or 60 min (CB_2_R) at 30°C. Non-specific binding was determined in the presence of 1 μM WIN-55,212-2. Bound and free radioactivity were separated by filtering the assay mixture as above indicated. The filter bound radioactivity was counted using a Packard Tri Carb 2810 TR scintillation counter (Perkin Elmer).

Competition binding experiments were also performed incubating 3 nM [^3^H]-WIN-55,212-2 and different concentrations of the tested compounds with membranes obtained from CHO cells transfected with human CB_1_ or CB_2_ receptors (10 μg protein/sample) for 60 min at 30°C. Non-specific binding was determined in the presence of 1 μM WIN-55,212-2. Bound and free radioactivity were separated by filtering the assay mixture as above indicated. The filter bound radioactivity was counted using a Packard Tri Carb 2810 TR scintillation counter (Perkin Elmer).

### Homogeneous Binding Assays in Living Cells

#### Expression Vector

cDNAs for the human version of cannabinoid CB_2_R without their stop codon were obtained by PCR and subcloned to SNAP-containing vector (PSNAP; Cisbio Bioassays) using sense and antisense primers harboring unique restriction sites for HindIII and BamHI generating the SNAP tagged CB_2_R (CB_2_R-SNAP).

#### Cell Culture and Transfection

For HTRF assays, HEK-293T cells were used. HEK 293T (HEK-293T) cells were grown in DMEM supplemented with 2 mM L-glutamine, 1 mM sodium pyruvate, 100 units/mL penicillin/streptomycin, and 5% (v/v) FBS [all supplements were from Invitrogen, (Paisley, Scotland, United Kingdom)]. Cells were maintained at 37°C in a humidified atmosphere of 5% CO_2_ and were passaged, with enzyme-free cell dissociation buffer (13151-014, Gibco^®^, Thermo Fisher, Waltham, MA, United States), when they were 80–90% confluent, i.e., approximately twice a week. Cells were transiently transfected with the PEI (Polyethylenimine, Sigma, St. Louis, MO, United States) method as previously described (Medrano et al., 2017; Navarro et al., 2018b). Experiments were carried out in cells expressing SNAP-tagged CB_2_R in the presence or in the absence of CB_1_R.

#### Labeling of Cells Expressing SNAP-Tagged CB_2_R

Cell culture medium was removed from the 25-cm^2^ flask and 100 nM SNAP-Lumi4-Tb, previously diluted in 3 mL of TLB 1X, was added to the flask and incubated for 1 h at 37°C under 5% CO_2_ atmosphere in a cell incubator. Cells were then washed four times with 2 mL of TLB 1X to remove the excess of SNAP-Lumi4-Tb, detached with enzyme-free cell dissociation buffer, centrifuged 5 min at 1,500 rpm and collected in 1 mL of TLB 1X. Tag-lite-based binding assays were performed 24 h after transfection. Densities in the 2,500–3,000 cells/well range were used to carry out binding assays in white opaque 384-well plates.

#### Non-radioactive Competition Binding Assays

For competition binding assays, the fluorophore-conjugated CB_2_R ligand (labeled CM-157), unconjugated CM-157 and CBG were diluted in TLB 1X. HEK-293T cells transiently expressing Tb-labeled SNAP-CB_2_R with or without CB_1_R were incubated with 20 nM fluorophore-conjugated CB_2_R ligand, in the presence of increasing concentrations (0–10 μM range) of CBG or CM-157. Plates contained 10 μL of labeled cells, and 5 μL of TLB 1X or 5 μL of CBG or 5 μL CM-157 were added prior to the addition of 5 μL of the fluorescent ligand. Plates were then incubated for at least 2 h at room temperature before signal detection. Detailed description of the HTRF assay is found in Martínez-Pinilla et al. (2016).

Signal was detected using an EnVision microplate reader (PerkinElmer, Waltham, MA, United States) equipped with a FRET optic module allowing donor excitation at 337 nm and signal collection at both 665 and 620 nm. A frequency of 10 flashes/well was selected for the xenon flash lamp excitation. The signal was collected at both 665 and 620 nm using the following time-resolved settings: delay, 150 μs; integration time, 500 μs. HTRF^®^ ratios were obtained by dividing the acceptor (665 nm) by the donor (620 nm) signals and multiplying by 10,000. The 10,000-multiplying factor is used solely for the purpose of easier data handling.

### Functional Assays

#### Cell Culture and Transient Transfection

HEK-293T cells were grown in DMEM medium (Gibco, Paisley, Scotland, United Kingdom) supplemented with 2 mM L-glutamine, 100 U/mL penicillin/streptomycin, MEM Non-Essential Amino Acids Solution (1/100) and 5% (v/v) heat inactivated Foetal Bovine Serum (FBS) (Invitrogen, Paisley, Scotland, United Kingdom). Cells were maintained in a humid atmosphere of 5% CO_2_ at 37°C. Cells were transiently transfected with the PEI (Polyethylenimine, Sigma, St. Louis, MO, United States) method as previously described (Medrano et al., 2017; Navarro et al., 2018b) and used for functional assays 48 h later (unless otherwise stated).

#### cAMP Determination

Signaling experiments have been performed as previously described (Navarro et al., 2010, 2016, 2018b; Hinz et al., 2018). Two hours before initiating the experiment, HEK-293T cell-culture medium was replaced by serum-starved DMEM medium. Then, cells were detached, resuspended in growing medium containing 50 μM zardaverine and placed in 384-well microplates (2,500 cells/well). Cells were pretreated (15 min) with CBG -or vehicle- and stimulated with agonists (15 min) before adding 0.5 μM forskolin or vehicle. Readings were performed after 15 min incubation at 25°C. HTRF energy transfer measures were performed using the Lance Ultra cAMP kit (PerkinElmer, Waltham, MA, United States). Fluorescence at 665 nm was analyzed in a PHERAstar Flagship microplate reader equipped with an HTRF optical module (BMG Lab Technologies, Offenburg, Germany).

#### ERK Phosphorylation Assays

To determine ERK1/2 phosphorylation, 50,000 HEK-293T cells/well were plated in transparent Deltalab 96-well microplates and kept at the incubator for 24 h. 2 to 4 h before the experiment, the medium was substituted by serum-starved DMEM medium. Then, cells were pre-treated at 25°C for 10 min with vehicle or CBG in serum-starved DMEM medium and stimulated for an additional 7 min with the specific agonists. Cells were then washed twice with cold PBS before addition of lysis buffer (20 min treatment). 10 μL of each supernatant were placed in white ProxiPlate 384-well microplates and ERK 1/2 phosphorylation was determined using AlphaScreen^®^SureFire^®^ kit (Perkin Elmer) following the instructions of the supplier and using an EnSpire^®^ Multimode Plate Reader (PerkinElmer, Waltham, MA, United States).

#### Dynamic Mass Redistribution Assays (DMR)

Cell mass redistribution induced upon receptor activation was detected by illuminating the underside of a biosensor with polychromatic light and measuring the changes in the wavelength of the reflected monochromatic light. The magnitude of this wavelength shift (in picometers) is directly proportional to the amount of DMR. HEK-293T cells were seeded in 384-well sensor microplates to obtain 70–80% confluent monolayers constituted by approximately 10,000 cells per well. Previous to the assay, cells were washed twice with assay buffer (HBSS with 20 mM HEPES, pH 7.15) and incubated for 2 h with assay-buffer containing 0.1% DMSO (24°C, 30 μL/well). Hereafter, the sensor plate was scanned and a baseline optical signature was recorded for 10 min before adding 10 μL of CBG for 30 min followed by the addition of 10 μL of specific agonists; all test compounds were dissolved in assay buffer. The cell signaling signature was determined using an EnSpire^®^ Multimode Plate Reader (PerkinElmer, Waltham, MA, United States) by a label-free technology. Then, DMR responses were monitored for at least 5,000 s. Results were analyzed using EnSpire Workstation Software v 4.10.

#### β-Arrestin 2 Recruitment

Arrestin recruitment was determined as previously described (Medrano et al., 2017; Navarro et al., 2018b). Briefly, BRET experiments were performed in HEK-293T cells 48 h after transfection with the cDNA corresponding to the CB_2_R-YFP or CB_1_R-YFP and 1 μg cDNA corresponding to β-arrestin 2-Rluc. Cells (20 μg protein) were distributed in 96-well microplates (Corning 3600, white plates with white bottom) and were incubated with CBG for 15 min and stimulated with the agonist for 10 min prior the addition of 5 μM coelenterazine H (Molecular Probes, Eugene, OR, United States). After 1 min of adding coelenterazine H, BRET between β-arrestin 2-Rluc and receptor-YFP was determined and quantified. The readings were collected using a Mithras LB 940 (Berthold Technologies, Bad Wildbad, Germany) that allows the integration of the signals detected in the short-wavelength filter at 485 nm and the long-wavelength filter at 530 nm. To quantify protein-RLuc expression luminescence readings were also performed 10 min of adding 5 μM coelenterazine H.

### Data Handling and Statistical Analysis

Affinity values (*K*i) were calculated from the IC_50_ obtained in competition radioligand binding assays according to the Cheng and Prusoff equation: *K*_i_ = IC_50_/(1 + [*C*]/*K*_D_), where [*C*] is the free concentration of the radioligand and *K*_D_ its dissociation constant (Cheng, 2001).

Data from homogeneous binding assays were analyzed using Prism 6 (GraphPad Software, Inc., San Diego, CA, United States). *K*_i_ values were determined according to the Cheng and Prusoff equation with *K*_D_ = 21 nM for CM-157 (Cheng, 2001). Signal-to-background (*S*/*B* ratio) calculations were performed by dividing the mean of the maximum value (μ_max_) by that of the minimum value (μ_min_) obtained from the sigmoid fits.

The data are shown as the mean ± SEM. Statistical analysis was performed with SPSS 18.0 software. The test of Kolmogorov–Smirnov with the correction of Lilliefors was used to evaluate normal distribution and the test of Levene to evaluate the homogeneity of variance. Significance was analyzed by one-way ANOVA, followed by Bonferroni’s multiple comparison *post hoc* test. Significant differences were considered when *p* < 0.05.

## Results

### Saturation and Competition Radioligand-Based Assays in Membranes Expressing CB_1_R or CB_2_R

**FIGURE 1:**
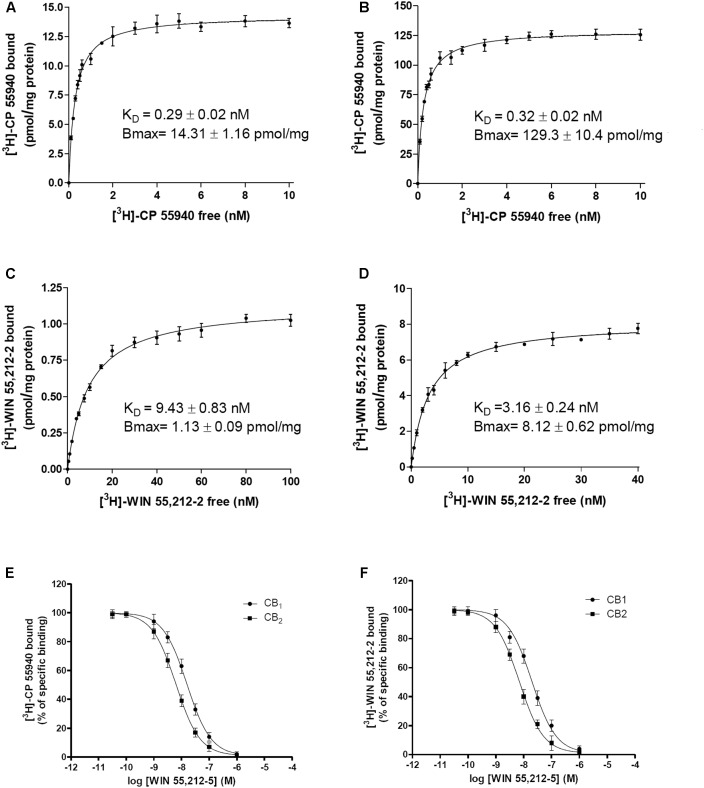
Radioligand binding assays to CB_1_R and CB_2_R. **(A–D)** Saturation curves of either [^3^H]-CP-55940 or [^3^H]-WIN-55,212-2 binding on membranes from CHO cells stably expressing human CB_1_R **(A,C)** or CB_2_R **(B,D)**. **(E,F)** Competition curves for WIN-55,212-2 in radioligand-based assays using either [^3^H]-CP-55940 **(E)** or [^3^H]-WIN-55,212-2 **(F)** binding on membranes from CHO cells stably expressing human CB_1_R or CB_2_R. Data are expressed as the mean ± SEM of five independent experiments performed in duplicate. *K*_D_ (obtained from saturation isotherms) are shown in **Table 1**.

The effect of CBG on radioligand binding to CB_1_R or CB_2_R was first tested using the classical radioligand-binding assay in membranes isolated from CHO cells expressing human CB_1_R or CB_2_R and incubated with radioligands: [^3^H]-CP-55940 or [^3^H]-WIN-55,212-2. Data obtained from binding isotherms using increasing [^3^H]-CP-55940 or [^3^H]-WIN-55,212-2 concentrations lead to a monophasic saturation curve. Saturation curves, receptor density (*B*_max_ values) and affinity (*K*_D_ values) are shown in **Figures 1A–D**. The affinity of the two radioligands was in the nanomolar range for both CB_1_R and CB_2_R. *K*_D_ for [^3^H]-CP-55940 to CB_1_R and CB_2_R was similar with values around 0.3 nM. *K*_D_ values for WIN-55,212-2 were 9.4 and 3.2 nM for CB_1_R and CB_2_R, respectively (**Figures 1C,D**). Overall the results agree with previously reported data (McPartland et al., 2007; Merighi et al., 2010).

**FIGURE 2:**
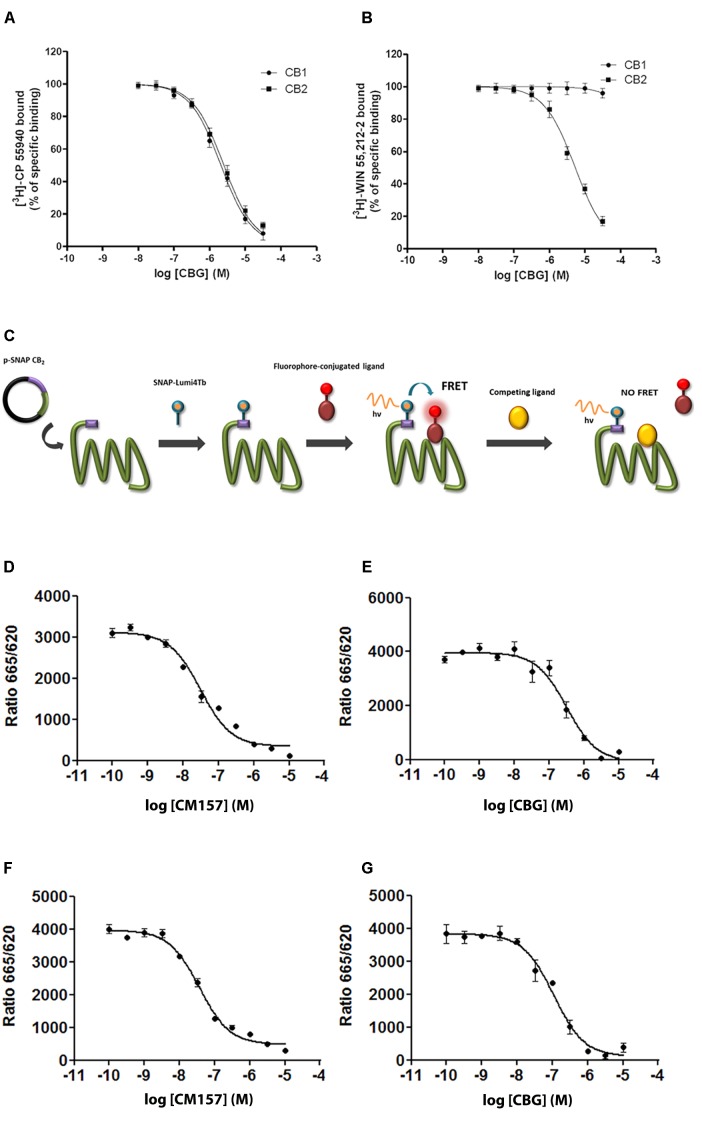
Competition by CBG of agonist binding to CB_1_R and/or CB_2_R. **(A,B)** Competition curves for CBG in radioligand-based assays using either [^3^H]-CP-55940 **(A)** or [^3^H]-WIN-55,212-2 **(B)** binding on membranes from CHO cells stably expressing human CB_1_R or CB_2_R. **(C)** Scheme of the HTRF-based competitive binding assay. The GPCR of interest with the SNAP-tagged enzyme fused to its N-terminal domain is expressed at the cell surface. SNAP is a commercially available tag consisting of circa 180 amino acids, that can be labeled with fluorophores or other probes in a covalent fashion. The GPCR–SNAP-tagged cells are subsequently labeled with a Tb-containing probe (SNAP-Lumi4-Tb) through a covalent bond between the Tb and the reactive side of the SNAP enzyme. The Tb acts as FRET donor of an acceptor covalently linked to a selective CB2 receptor ligand. Thus, upon binding of a fluorophore-conjugated ligand (FRET acceptor) on the donor-labeled SNAP-tagged/GPCR fusion protein, an HTRF signal from the sensitized acceptor can be detected since the energy transfer can occur only when the donor and the acceptor are in close proximity. In competition binding assays using CM-157, the unlabelled specific ligand competes for receptor binding site with the fluorophore-conjugated ligand, leading to a decrease in the HTRF signal detected. **(D–G)** HEK-293T were transiently transfected with 1 μg cDNA for SNAP-CB_2_R in the absence **(D,E)** or presence of 0.5 μg cDNA for CB_1_R **(F,G)**. Competition curves of specific binding of 20 nM fluorophore-conjugated CM-157 using CM-157 (0–10 μM) **(D,F)** or of CBG (0–10 μM) **(E,G)** as competitors are shown. Data represent the mean ± SEM of five experiments in triplicates.

Competition binding assays of WIN-55,212-2 showed similar *K*_i_ values using the two radioligands to CB_1_R and CB_2_R and agreed with the *K*_D_ values for [^3^H]-WIN-55,212-2 binding (**Table 1** and **Figures 1E,F**). **Table 1** reports the affinity values of CBG. *K*_i_ values of CBG obtained using [^3^H]-CP-55940 as radioligand were in the low micromolar range in both CB_1_R and CB_2_R. The affinity value of CBG obtained using [^3^H]-WIN-55,212-2 for CB_2_R was 2.7 μM, about twofold higher than that obtained using [^3^H]-CP-55940. Using [^3^H]-WIN-55,212-2 in competition binding experiments on CB_1_R, CBG was not able to displace the radioligand (**Figures 2A,B**). In summary, CBG displayed *K*_i_ values in the low micromolar range when competing for the binding to the CB_2_R. Surprisingly, significant competition in the binding to the CB_1_R was only observed when using [^3^H]-CP-55940 as radioligand.

**Table 1:** Affinity values of CB compounds obtained from radioligand binding assays.

	[^3^H]-CP-55940 competition binding experiments		[^3^H]-WIN-55,212-2 competition binding experiments	
		
	CB_1_ – *K*_D_ (nM)	CB_2_ – *K*_D_ (nM)	CB_1_ – *K*_D_ (nM)	CB_2_ – *K*_D_ (nM)
	0.29 ± 0.02	0.32 ± 0.02	9.43 ± 0.83	3.16 ± 0.24
	
	CB_1_ – *K*_i_ (nM)	CB_2_ – *K*_i_ (nM)	CB_1_ – *K*_i_ (nM)	CB_2_ – *K*_i_ (nM)
WIN-55,212-2	8.08 ± 0.65	3.22 ± 0.31	9.86 ± 0.84	3.48 ± 0.27
CBG	1,045 ± 74	1,225 ± 85	>30,000	2,656 ± 130
CBD	1,690 ± 110	1,714 ± 70	>30,000	4,019 ± 342


*K_*D*_ values were obtained from saturation isotherms and K_*i*_ from data in competition assays using the indicated radiolabelled compounds ([^*3*^H]-CP-55940 or [^*3*^H]-WIN-55,212-2).*

### CBG Binds to the Orthosteric Site of Cannabinoid CB_2_R at Nanomolar Concentrations

Competition experiments were performed using 20 nM of a fluorophore-conjugated selective CB_2_R agonist (CM-157) and a homogeneous non-radioactive method performed in living cells expressing SNAP-CB_2_R (details in Martínez-Pinilla et al., 2016; **Figure 2C**). Unfortunately, the equivalent fluorophore-conjugated selective CB_1_R ligand is not available to perform HTRF assays in SNAP-CB_1_R-expressing living cells. Competition assays were performed in HEK-293T cells expressing Lumi4-Tb-labeled CB_2_R fused to the SNAP protein and incubated with a fixed amount of the fluorophore-conjugated agonist and different CBG concentrations. As observed in **Figure 2**, both the unlabelled selective agonist (CM-157) and CBG decreased the binding to SNAP-CB_2_R in monophasic fashion and with *K*_i_ values in the nanomolar range (16 nM for CM-157 and of 152 nM for CBG; **Figures 2D,E**). The *K*_i_ obtained for CM-157 matches with previously reported dissociation constant *K*_D_ values (Martínez-Pinilla et al., 2016). These results indicate that CBG can significantly bind to the orthosteric site of cannabinoid CB_2_R at nanomolar concentrations.

Similar experiments were carried out in HEK-293T cells expressing SNAP-CB_2_R fusion protein and a similar amount of CB_1_R, i.e., in cells that express CB_2_R in a CB_1_–CB_2_ receptor heteromer context. In the presence of cannabinoid CB_1_R the *K*_i_ for CM-157 was 19 nM (**Figure 2F**) and *K*_i_ for CBG was reduced (56 nM, **Figure 2G**). These results indicate that in cells expressing both cannabinoid receptors, CB_1_ and CB_2_, CBG shows higher affinity for cannabinoid CB_2_R.

### CBG Effects on Cannabinoid Receptor-Agonist-Induced Effects

**FIGURE 3:**
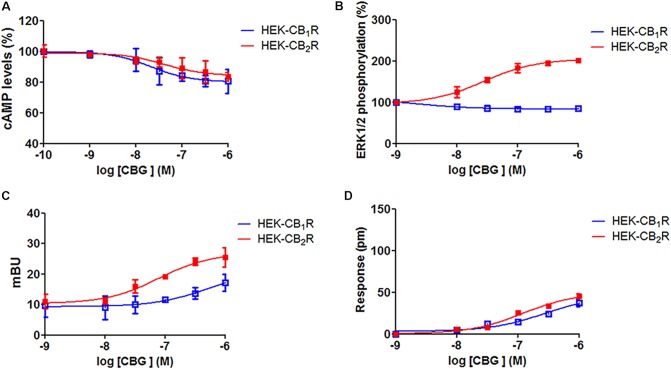
Cannabigerol action in cells expressing CB_1_R or CB_2_R. HEK-293T cells were transfected with 0.75 μg cDNA for CB_1_R (red line) or 1 μg cDNA for CB_2_R (blue line). Dose–effect curves for cAMP production are expressed as % of levels obtained by 0.5 μM forskolin treatment **(A)**. Dose-effect curves for ERK1/2 phosphorylation are expressed as % respect to basal levels **(B)**. Dose-effect curves for β-arrestin recruitment **(C)** and label-free **(D)** assays are expressed, respectively, in mBRET units and pm. In β-arrestin-2 recruitment assays cells were transfected with 1 μg cDNA for β-arrestin-Rluc and either 0.75 μg cDNA for CB_1_R-YFP or 1 μg cDNA for CB_2_R-YFP. Data are the mean ± SEM of a representative experiment in triplicates (*n* = 6).

Previous reports Gauson et al. (2007), Cascio et al. (2010) suggest that CBG may be a partial agonist of cannabinoid receptors. To investigate this possibility, HEK-293T cells expressing CB_1_R or CB_2_R were treated with increasing concentrations of CBG (1 nM to 10 μM) and cAMP, MAPK, β-arrestin recruitment and dynamic mass cell redistribution (DMR) assays were developed. Interestingly, it was observed that in cells expressing CB_1_R (**Figure 3**, blue curves), CBG induced a small decrease in forskolin induced cAMP levels and a small increase in β-arrestin recruitment (**Figures 3A,C**), while having no significant action on MAPK phosphorylation assay (**Figure 3B**). Consequently, CBG in label-free assays induced a slight effect in the DMR signal (**Figure 3D**) that is consistent with a G protein-dependent action on cAMP levels; label-free signal is based on optical detection of DMR following receptor activation and mainly reflects G-protein-coupling (Kebig et al., 2009; Schröder et al., 2009; Hamamoto et al., 2015). On the other hand, in HEK-293T cells expressing CB_2_R (**Figure 3**, red curves), the action on forskolin-induced cAMP levels and on the DMR signal was small and similar to that exerted in CB_1_R-expressing cells (**Figure 3A**). On the contrary, the activation of the MAP kinase pathway was notable (**Figure 3B**). Also noteworthy was the CBG-induced β-arrestin recruitment (**Figure 3C**). Taken together these data suggest that CBG is a poor agonist of CB_1_R, whereas it acts as a partial agonist in some of the signaling pathways analyzed in cells expressing CB_2_R.

**FIGURE 4:**
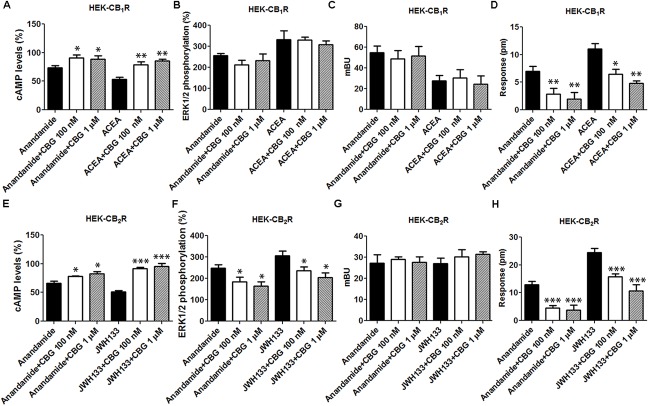
Effect of CBG on the action of CB_1_R and CB_2_R agonists. **(A–D)** HEK-293T cells were transfected with 0.75 μg cDNA for CB_1_R and treated with 100 nM AEA or a selective CB_1_R ligand (100 nM ACEA) in the absence (black bars) or presence of 100 nM (white bars) or 1 μM (gray bars) CBG. **(E–H)** HEK-293T cells were transfected with 1 μg cDNA for CB_2_R and treated with 100 nM AEA or a selective CB_2_R ligand (100 nM JWH133) in the absence (black bars) or presence of 100 nM (white bars) or 1 μM (gray bars) CBG. cAMP production **(A,E)** is expressed as % of levels obtained by 0.5 μM forskolin. ERK1/2 phosphorylation data are expressed as % respect to basal levels **(B,F)**. In β-arrestin-2 recruitment assays cells were transfected with 1 μg cDNA for β-arrestin-Rluc and either 0.75 μg cDNA for CB_1_R-YFP or 1 μg cDNA for CB_2_R-YFP. Data for β-arrestin recruitment **(C,G)** and label-free **(D,H)** assays are expressed, respectively, in mBRET units and pm. Data represent the mean ± SEM of six different experiments performed with six replicates. One-way ANOVA and Bonferroni’s multiple comparison *post hoc* test were used for statistical analysis (^∗^*p* < 0.05, ^∗∗^*p* < 0.01, ^∗∗∗^*p* < 0.001; versus treatment with AEA, ACEA, or JWH133 alone).

To further examine the CBG effect over CB_1_R, HEK-293T cells expressing CB_1_R were treated with the endocannabinoid agonist, AEA, or with ACEA in the presence or in the absence of 100 nM or 1 μM CBG. In forskolin-induced cAMP assays we found that 100 nM or 1 μM CBG pretreatment induced a significant decrease in both, AEA and ACEA induced effects (**Figure 4A**). In contrast, CBG (100 nM or 1 μM) was unable to modify the agonist-induced MAPK phosphorylation and β-arrestin recruitment (**Figures 4B,C**). In label-free DMR assays the results were similar to those obtained in cAMP determination assays, i.e., CBG reduced the effect of the agonists (**Figure 4D**).

Cannabigerol (100 nM or 1 μM) was also tested in HEK-293T cells expressing CB_2_R and using AEA and a receptor selective agonist, JWH133. Pretreatment with CBG reduced the effects of AEA and JWH133 in experiments of forskolin-induced cAMP levels, ERK1/2 phosphorylation and in label-free DMR read-outs (**Figure 4**). In contrast, CBG did not affect the recruitment of β-arrestin induced by agonists (**Figure 4G**). This last result may be due to the low sensitivity of the assay as β-arrestin recruitment BRET signal was virtually negligible. Energy transfer techniques completely depend on the correct orientation of the fusion proteins and the reduced signal may be due to poor recruitment of β-arrestin and/or to a high distance between BRET donor/acceptor in the putative β-arrestin-Rluc/CB_2_R-YFP complex. Thus, CBG in cells activated by endocannabinoids or by selective agonists behaves as a partial agonist of the CB_2_R.

### CBG Effect in HEK-293T Cells Expressing CB_1_R and CB_2_R

**FIGURE 5:**
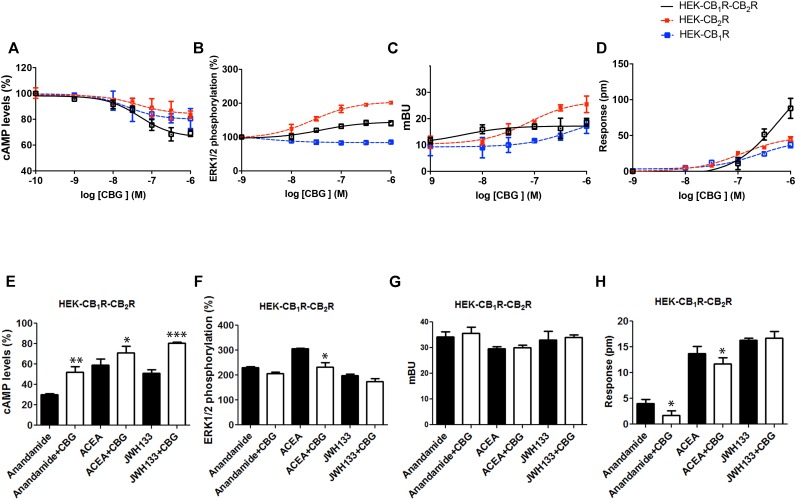
Effect of CBG in cells expressing CB_1_ and CB_2_ receptors. **(A–D)** Effect of CBG in HEK-293T cells transfected with 0.75 μg cDNA for CB_1_R and 1 μg cDNA for CB_2_R **(A,B,D)** or 1 μg cDNA for β-arrestin-Rluc, 0.75 μg cDNA for CB_1_R and 1 μg cDNA for CB_2_R-YFP **(C)**. Dose–effect curves for cAMP production are expressed as % of levels obtained by 0.5 μM forskolin treatment **(A)**. Dose-effect curves for ERK1/2 phosphorylation are expressed as % respect of basal levels **(B)**. Dose-effect curves for β-arrestin recruitment **(C)** and label-free **(D)** assays are expressed, respectively, in mBRET units and pm. Dotted lines (red and blue) are the same than those shown in **Figure 3** and serve as a reference for differential effects in cells coexpressing both receptors. Data are the mean ± SEM of a representative experiment in triplicates (*n* = 6). **(E–H)** HEK-293T cells transfected with 0.75 μg cDNA for CB_1_R and 1 μg cDNA for CB_2_R **(E,F,H)** or 1 μg cDNA for β-arrestin-Rluc, 0.75 μg cDNA for CB_1_R and 1 μg cDNA for CB_2_R-YFP **(G)** were treated with 100 nM AEA or a selective CB_2_R ligand (100 nM JWH133) in the absence (black bars) or presence (white bars) of 100 nM CBG. cAMP production **(E)** is expressed as % of levels obtained by 0.5 μM forskolin. ERK1/2 phosphorylation data are expressed as % respect of basal levels **(F)**. Data for β-arrestin recruitment **(G)** and label-free **(H)** assays are expressed, respectively, in mBRET units and pm. Data represent the mean ± SEM of six different experiments performed with three replicates. One-way ANOVA and Bonferroni’s multiple comparison *post hoc* test were used for statistical analysis (^∗^*p* < 0.05, ^∗∗^*p* < 0.01, ^∗∗∗^*p* < 0.001; versus treatment with AEA, ACEA, or JWH133 alone).

Experiments were finally performed in cells co-expressing the two cannabinoid receptors, which are able to form heteromeric complexes. A CB_1_–CB_2_ receptor heteromer print consists of a negative cross-talk observed in Akt phosphorylation and neurite outgrowth; i.e., activation of one receptor reduces the signaling originated upon partner receptor activation (Callén et al., 2012). To characterize the CBG effect, experiments were performed in HEK-293T cells expressing the two cannabinoid receptors. Dose-effect curves were provided for cAMP level and ERK1/2 phosphorylation determination, and for label-free DMR signal and β-arresting recruitment. Interestingly, the effect on cAMP level determination and DMR assays was additive (**Figure 5**), i.e., the presence of CBG blunted the negative cross-talk in these signaling pathways. However, the negative cross-talk was still evident in both ERK1/2 phosphorylation and β-arrestin recruitment experiments (**Figures 5B,C**).

Finally, the effect of 100 nM CBG (100 nM) on AEA, ACEA and/or JWH133 actions was investigated in cells co-expressing CB_1_R and CB_2_R. CBG pretreatment led to significant effects, always reducing the effect of the agonists, in cAMP-related assays (**Figure 5E**). However, the effect in the other assay types was negligible except for the negative modulation of the ACEA effect on ERK1/2 phosphorylation and DMR, and of the AEA effect on DMR read-outs (**Figures 5F–H**). Therefore, CBG either blunted the cAMP-dependent signaling or did not significantly alter the negative cross-talk when other CB_1_/CB_2_-mediated signaling read-outs were determined (see **Figures 5B,C**). It should be noted that cross-talk at the intracellular signaling level, cannot be ruled out to partly explain some of the findings (Bayewitch et al., 1995; Wartmann et al., 1995; Mcguinness et al., 2009; Peters and Scott, 2009; Van Der Lee et al., 2009).

## Discussion

The aim of this paper was to comparatively address CBG pharmacology and effects on CB_1_ and CB_2_ receptors, and on CB_1_–CB_2_ heteroreceptor complexes. The binding experiments using radiolabelled- and non-radiolabelled-based approaches have provided relevant results. The results on CB_2_R are clear an indicate that CBG acts as a competitive partial agonist ligand. There is, however, an interesting observation as the *K*_i_ values for competing both [^3^H]-CP-55940 and [^3^H]-WIN-55,212-2 are in the low micromolar range (**Table 1**), whereas displaying a value of 152 nM in HTRF-based assays. As pointed out in previous reports, the conditions of the approach using a fluorescent-conjugated CM-157 allows identification of different states of the receptor. Irrespective of the molecular mechanism, the marked differences in affinity constants suggest different ways to accommodate the ligand within the orthosteric center. To our knowledge this is the first report performed in parallel binding assays using three different ligands that reportedly bind to the orthosteric center of the CB_2_R ([^3^H]-CP-55940, [^3^H]-WIN-55,212-2 and fluorescence-conjugated-CM-157). In summary, the most reasonable assumption is that CBG binds to the orthosteric center of CB_2_R but with marked differences in affinity depending on the assay. It should be noted that differences in affinity may result from the fact that HTRF binding is performed in living cells whereas radioligand binding assays are performed in isolated membranes. The already existing data concerning CBG affinity for CB_1_ and CB_2_ receptors, all performed using [^3^H]-CP-55940 also indicate that the affinity may vary depending on the context of the receptor, by *inter alia* the constraints of the membrane, heteromerization or interaction with G-proteins. Comparing our results with similar data using [^3^H]-CP-55940, the affinity is higher for receptors expressed in HEK-293 cells or in brain membranes (Gauson et al., 2007; Pertwee, 2008; Pollastro et al., 2011) that in receptors expressed in CHO cells (**Table 1**). In competition assays of radioligand binding to CB_1_R or to CB_2_R, affinity for CBG is similar to that previously published (Gauson et al., 2007; Pertwee, 2008), except in the case of Sf9 cells (*K*_i_: 897 and 153 nM for, respectively, CB_1_R and CB_2_R). This piece of data would indicate conformational changes induced by third molecules that affect the binding of the radioligand and/or of CBG. In fact, Sf9 are insect cells that do not express the cognate G_i_ protein and, therefore, Gαi3β1γ2 was heterologously expressed to perform the binding assays that led to different affinities for CBG (897 and 153 nM for, respectively, CB_1_R and CB_2_R) (Rosenthaler et al., 2014).

The results from binding to the CB_1_R are not very robust and more difficult to interpret. Unfortunately, there are no ligands available to perform HTRF binding to SNAP-CB_1_R-expressing living cells, whereas the data from competition assays using [^3^H]-CP-55940 or [^3^H]-WIN-55,212-2 were contradictory. On the one hand, the *K*_i_ for binding to the CB_1_R using [^3^H]-CP-55940 was in the low micromolar range, as it occurred with data from radioligand binding to the CB_2_R. However, CBG was unable to compete [^3^H]-WIN-55,212-2 binding to the CB_1_R. Taking into account that recognition sites for CP-55940 and WIN-55,212-2 are not identical in the CB_1_R, one possibility is that CBG binds to the orthosteric center but displaying different equilibrium binding parameters depending on the radioligand. It was early observed that Lys^192^ in the CB_1_R third transmembrane domain (TM3) was crucial for binding of CP-55940 and AEA but not for WIN-55,212-2 (Bonner et al., 1996; Chin et al., 1998). Later, *in silico* models pointed to an hydrophobic pocket for CP-55940 binding that involved residues in different transmembrane domains (not only in TM3) and in the second extracellular loop (Shim et al., 2003). Those models showed that WIN-55,212-2 not only binds to the hydrophobic pocket described for CP-55940 but to another hydrophobic region involving residues in TM2 and TM3 (Shim and Howlett, 2006). The structure of CBG is more similar to CP-55940 than to WIN-55,212,2, bearing an OH in the A ring that may interact with the TM3 Lys^192^ residue. In brief, CBG binds to the orthosteric center of CB_1_R as indicated by the fact that CBG affects CP-55940 binding without affecting the binding of [^3^H]-WIN-55,212-2. In other words, CBG was able to distinguish between two subregions of the CB_1_R orthosteric center. We therefore suggest that pharmacological studies concerning the CB_1_R should be run in parallel using radiolabelled CP-55940 and WIN-55,212-2. Interestingly CP-55940 and WIN-55,212-2 are able to fix the CB_1_R in two different conformations (Georgieva et al., 2008) and, therefore, CBG would affect more the conformation and signaling arising from occupation of the CP-55940 binding site. Other possibilities cannot be ruled out and, in this respect, we assayed CBD in competition assays and obtained similar results than those obtained using CBG (**Table 1**). Accordingly, CBG could act on CB_1_R (but not on CB_2_R) as non-competitive (allosteric) modulator, as described for CBD (Laprairie et al., 2015).

When one compound binds to the orthosteric center and affects several signaling pathways with different potency as in the case of CBG in cells expressing CB_1_R, the phenomenon is known as functional selectivity or biased agonism. In cells expressing CB_1_R, CBG effect is skewed toward the G_i_-mediated signaling pathway. This is in agreement with our finding of significant effect in label-free assays; often DMR signals correlate with effect on cAMP levels in the case of receptors coupled to G_i_ or G_s_ proteins (Grundmann and Kostenis, 2015a,b; Hamamoto et al., 2015). It is, however, intriguing that CBG was unable to displace the binding of [^3^H]-WIN-55,212-2 to the CB_1_R. Therefore, an action of CBG on a particular state of the receptor, which, in the case of CB_2_R may be disclosed by HTRF binding in living cells (Martínez-Pinilla et al., 2016), cannot be ruled out. Taking together all results, an allosteric action of CBG on the CB_1_R would not explain why it is able to engage G_i_ –mediating signaling. Another possibility, which was suggested for AM630, a previously considered CB_2_R antagonist (Bolognini et al., 2012), is that CBG is a protean agonist displaying biased agonism.

Data from CB_2_R-mediated functional assays were easier to interpret. First of all, the efficacy was lower compared to selective synthetic agonists and endocannabinoids. Also, CBG led to biased agonism as the effect on cAMP levels was small while being quite marked in ERK phosphorylation and β-arrestin recruitment. Therefore, CBG acted as a partial agonist and, as such, it was able to reduce the effects of other cannabinoid agonists. At 1 μM the effect of CBG on receptor activation by other agonists was similar to that exerted by 100 nM (**Figure 4**) thus suggesting that the *effective* affinity in living cells is that obtained in HTRF non-radioactive-based assays.

Due to the complex pharmacology of cannabinoids this research was undertaken to investigate whether CBG could be exerting a differential action on the CB_1_–CB_2_ receptor heteromers. Previous data have shown that the interplay between the two receptors in an heteromeric context is also complex. Whereas Callén et al. (2012) showed a negative cross-talk in a heterologous expression system, the allosteric interaction in the CB_1_–CB_2_ heteroreceptor complex is synergistic in primary cultures of activated microglia activated with LPS and interferon gamma and in primary cultures of microglia from a transgenic model of Alzheimer’s disease (Navarro et al., 2018a). Dose-effect experiments here undertaken in the HEK-293T-based heterologous expression system showed that CBG treatment in the absence of any other agonist, led to additive/synergistic effects on cAMP and label-free read-outs. In contrast, in ERK phosphorylation and β-arrestin recruitment, we found the negative cross-talk already described for this heteromer when full agonists are used to activate the receptors (Callén et al., 2012). These results suggest that partial agonism on the CB_2_R is regulated by the presence of CB_1_R; however, more complex alternative scenarios cannot be ruled out as CBG may act on the orthosteric site of the CB_2_R protomer and as protean agonist of the CB_1_R protomer. In cells expressing the two receptors, the overall effect of 100 nM CBG on agonist-induced activation is more consistent with acting on CB_2_R than on CB_1_R. In fact, the results in co-expressing cells, which likely express heteromers, are similar to those encountered in CB_2_R-expressing cells. In summary, CBG significantly modulates CB_2_R- or CB_1_R/CB_2_R-mediated endocannabinoid action, while the effects are weak in CB_1_R-expressing cells. Our findings demonstrating the action of CBG on the cannabinoid receptors are in complete agreement and may explain the *in vitro* results, reporting the protection of macrophages against oxidative stress (Giacoppo et al., 2017), and the beneficial *in vivo* effects in a model of inflammatory bowel disease (Borrelli et al., 2013). In the first of these two studies CBG-mediated protection is blocked by AM630, a selective CB_2_R ligand, whereas the CB_1_R antagonist, SR141716A, had no effect on CBG action (Giacoppo et al., 2017). The second study reported that CBG may both reduce the histological and molecular changes of experimental colitis and nitrite release from macrophages after LPS stimulation; again these effects were seemingly mediated by CB_2_R (Borrelli et al., 2013). These results can be explained by our findings; CBG acting as a partial agonist and exerting actions via CB_2_R in macrophages (Giacoppo et al., 2017) or “antagonizing” the effects of endogenous or synthetic cannabinoids, as in LPS-stimulated macrophages (Borrelli et al., 2013). In conclusion, the results presented in this study reveal that the non-psychotropic phytocannabinoid, CBG, may exert beneficial actions with therapeutic potential via cannabinoid receptors.

## Author Contributions

XN and RF had the original idea, designed and coordinated actions in the different participating institutions, and wrote the initial manuscript. GN performed non-radiolabelled-based homogeneous binding assays, participated in the signaling experiments, and significantly contributed to manuscript preparation. IR-R participated in the signaling experiments and in writing methods. RR-S actively participated in data analysis and parameter calculation. EC supervised data analysis, provided pharmacological expertise, and insight into data interpretation. FV performed the radioligand binding experiments. KV and PB performed the radioligand binding data analysis and interpretation. SC selected the *Cannabis* varieties and supervised the production of the vegetal raw material used for the isolation and purification of cannabinoids. VSM performed the isolation and purification of cannabinoids. CS-CC and CF-V performed the analytical quality control to the purified cannabinoids. All co-authors critically revised, contributed to the editing, and approved the manuscript.

## Conflict of Interest Statement

Authors declare that this research was undertaken in collaboration with Phytoplant Research S.L. Co-authors working in the Spanish and Italian public institutions do not receive honoraria from the company and do not have any participation in the company (stock shares or similar).

## Abbreviations

Δ^8^-THC: Δ^8^-tetrahydrocannabinol
Δ^9^-THC: Δ^9^-tetrahydrocannabinol
Δ^9^-THCA: Δ^9^- tetrahy drocannabinolic acid
Δ^9^-THCV: Δ^9^-tetrahydrocannabivarin
2-AG: 2-arachidonoyl glicerol
AEA: anandamide
CB_1_R: cannabinoid receptor 1
CB_2_R: cannabinoid receptor 2
CBC: cannabichromene
CBD: cannabidiol
CBDA: cannabidiolic acid
CBDV: cannabidivarin
CBG: cannabigerol
CBGA: cannabigerolic acid
CBN: cannabinol
CNS: central nervous system
DMR: dynamic mass redistribution
HEK: human embryonic kidney
HTRF: homogeneous time-resolved fluorescence
SNAP: protein used as a tag; it contains circa 180 amino acids and may be covalently labeled with different probes
Tb: terbium
TLB: Tag-lite labeling medium
